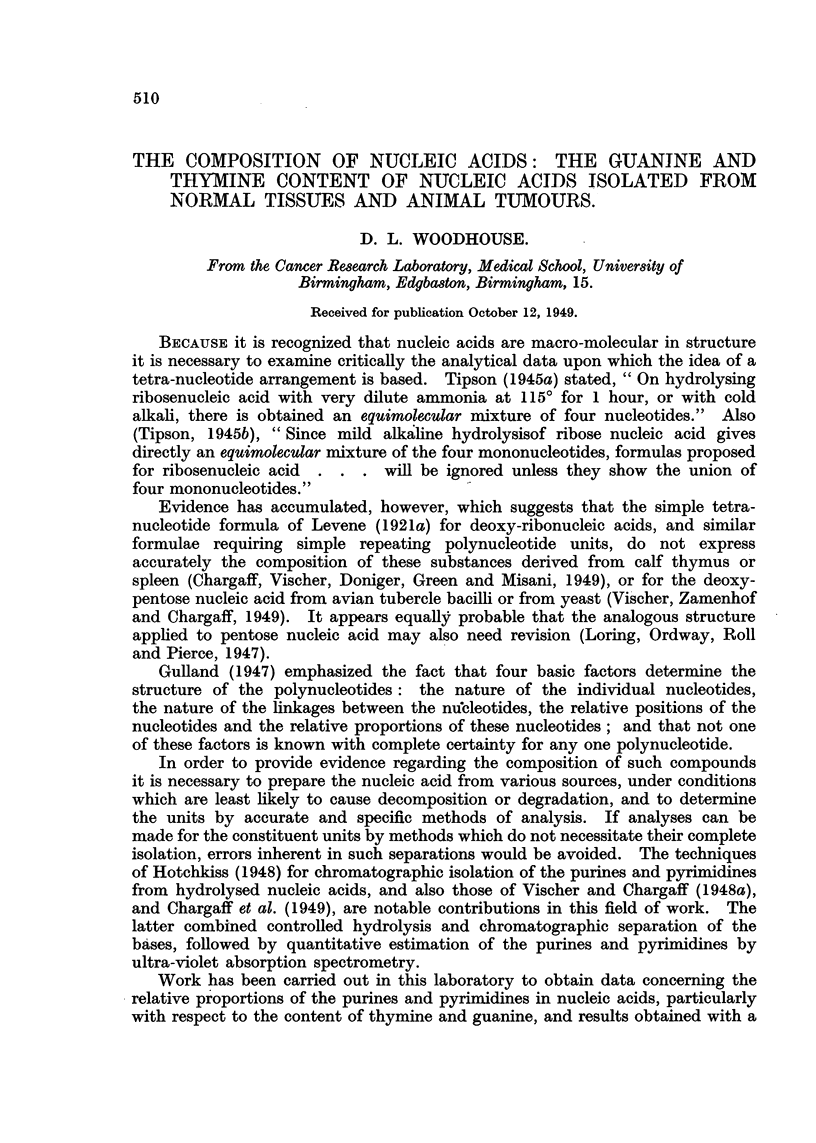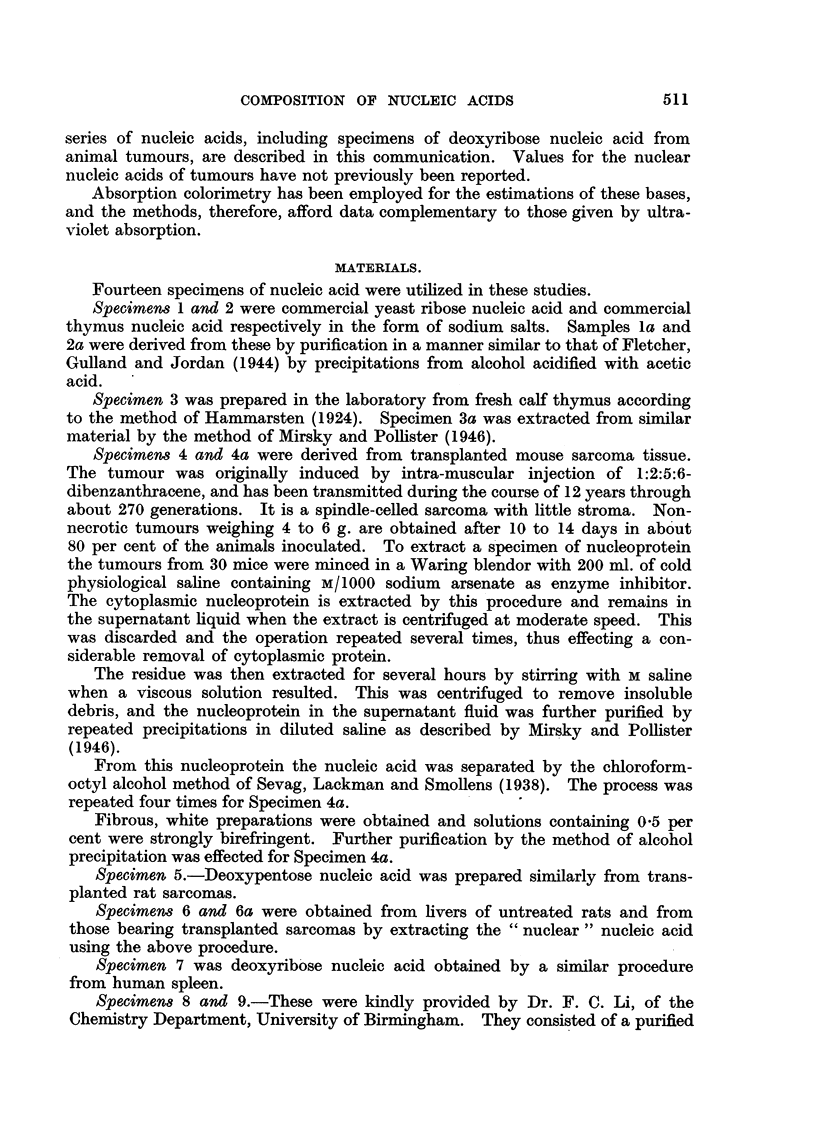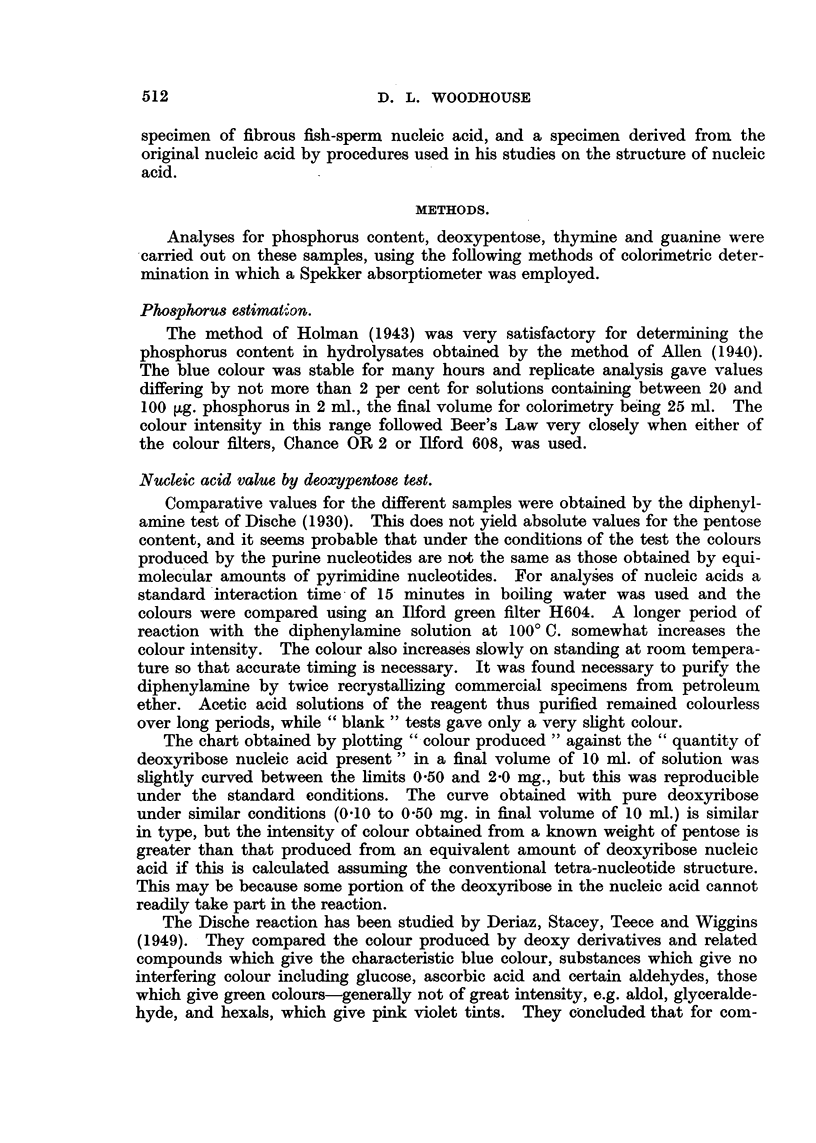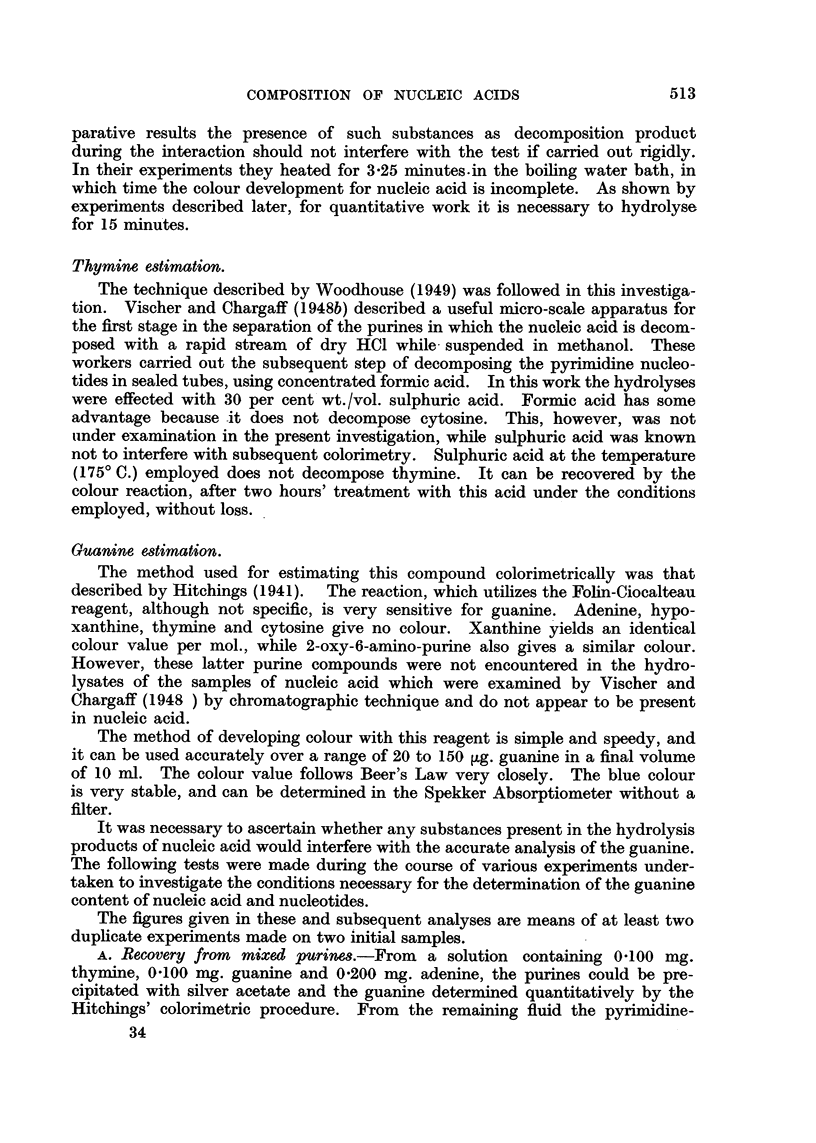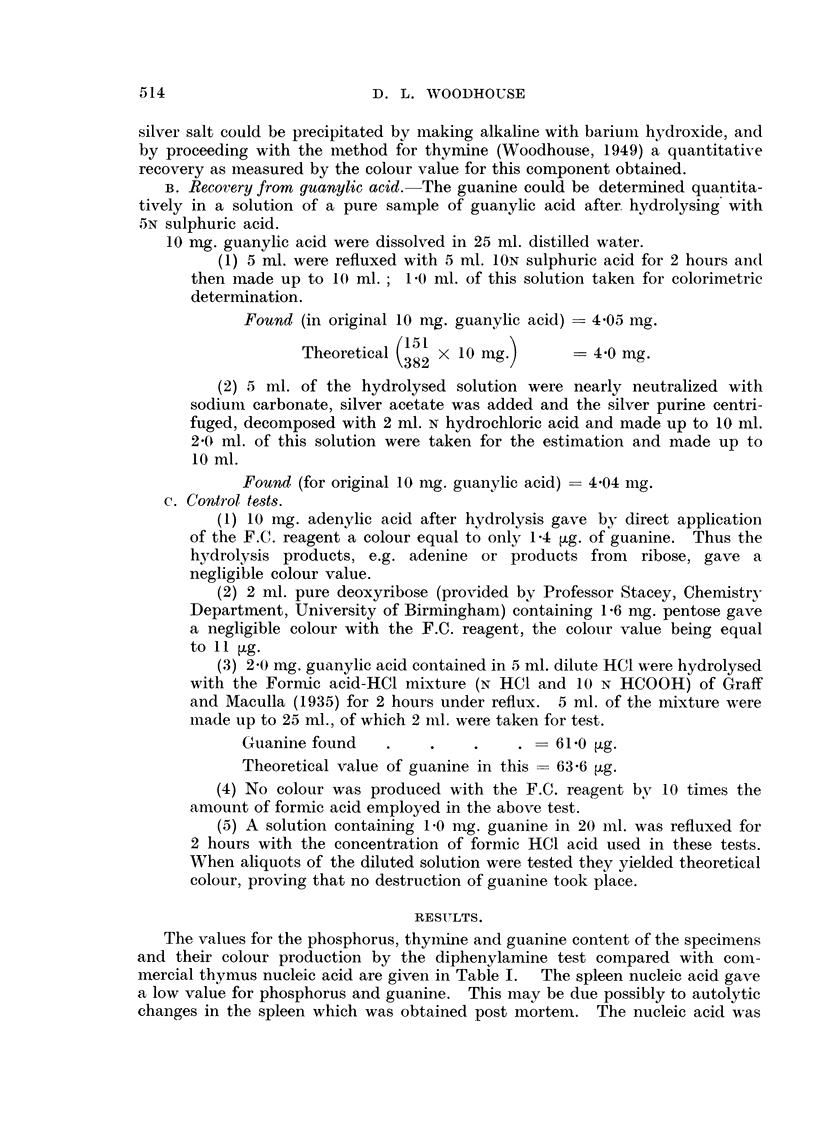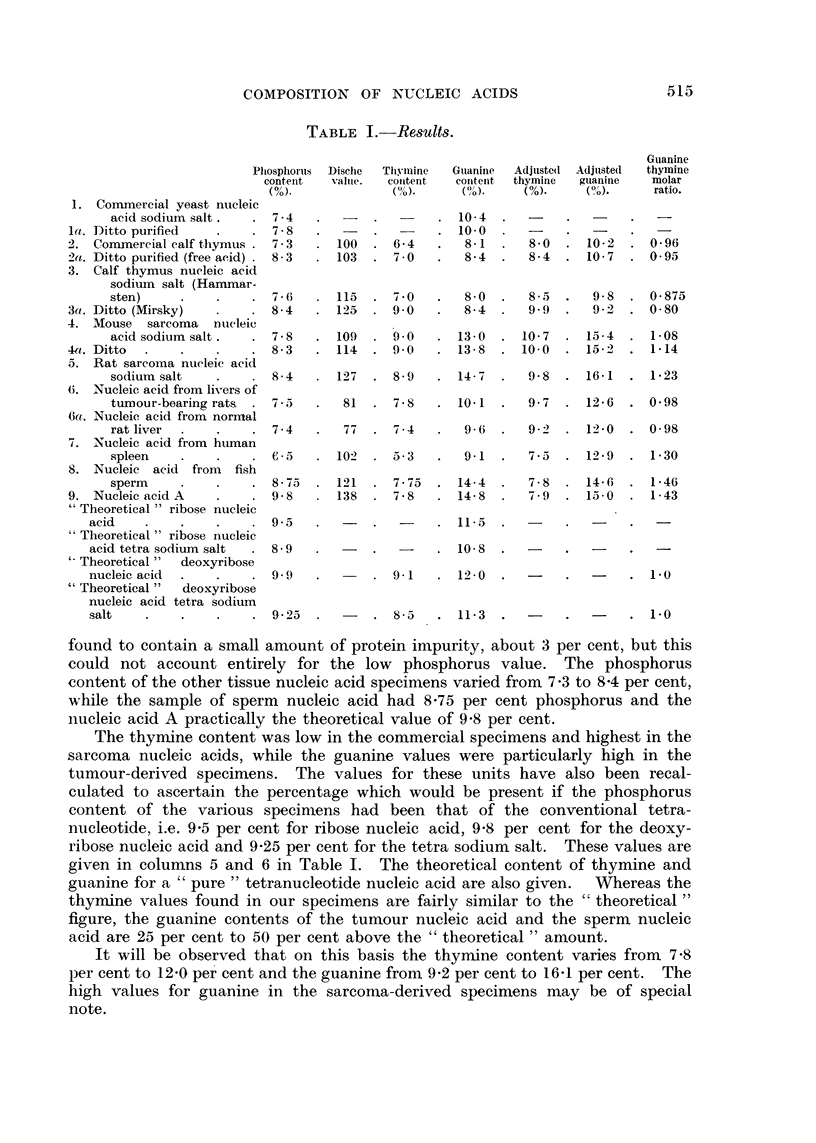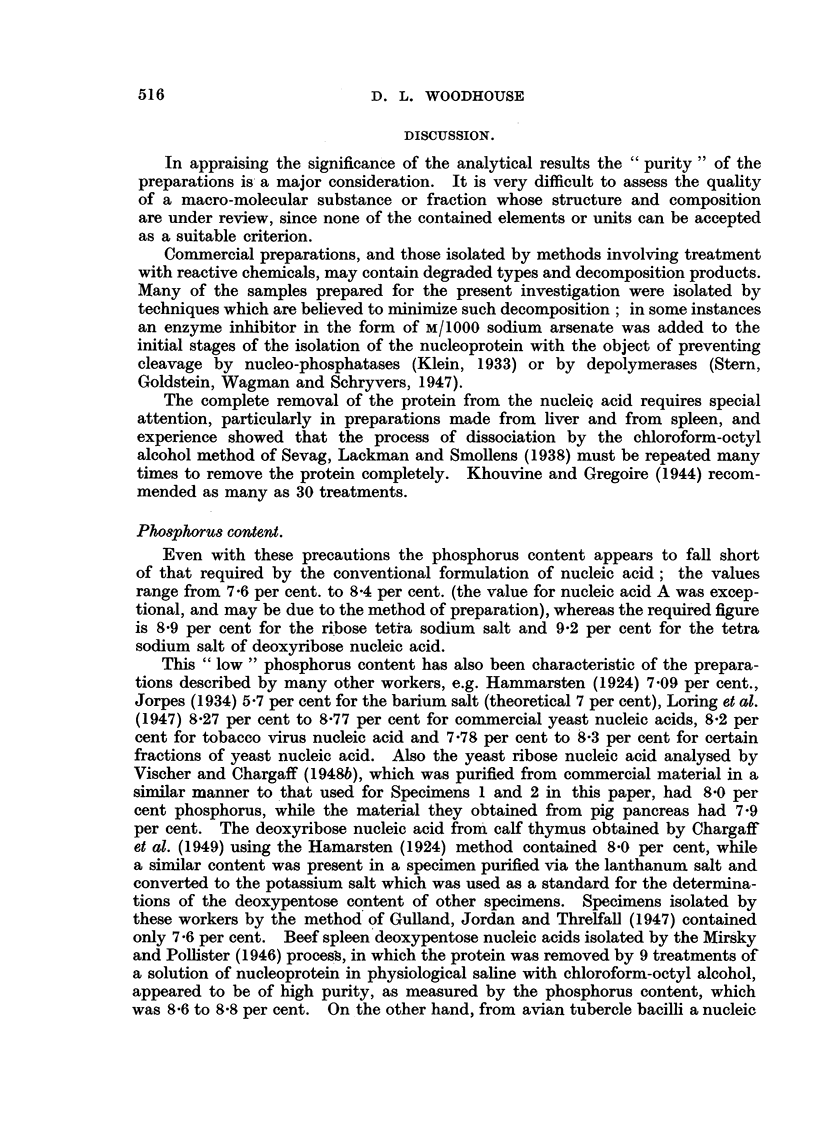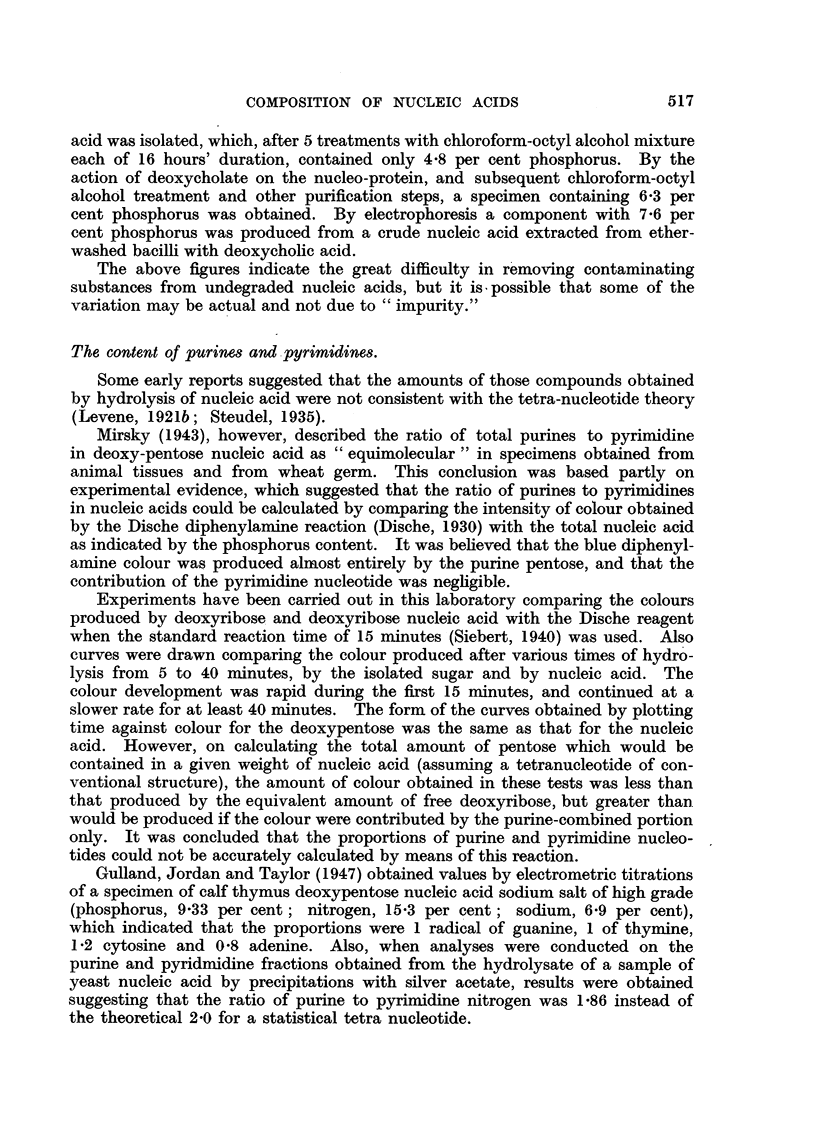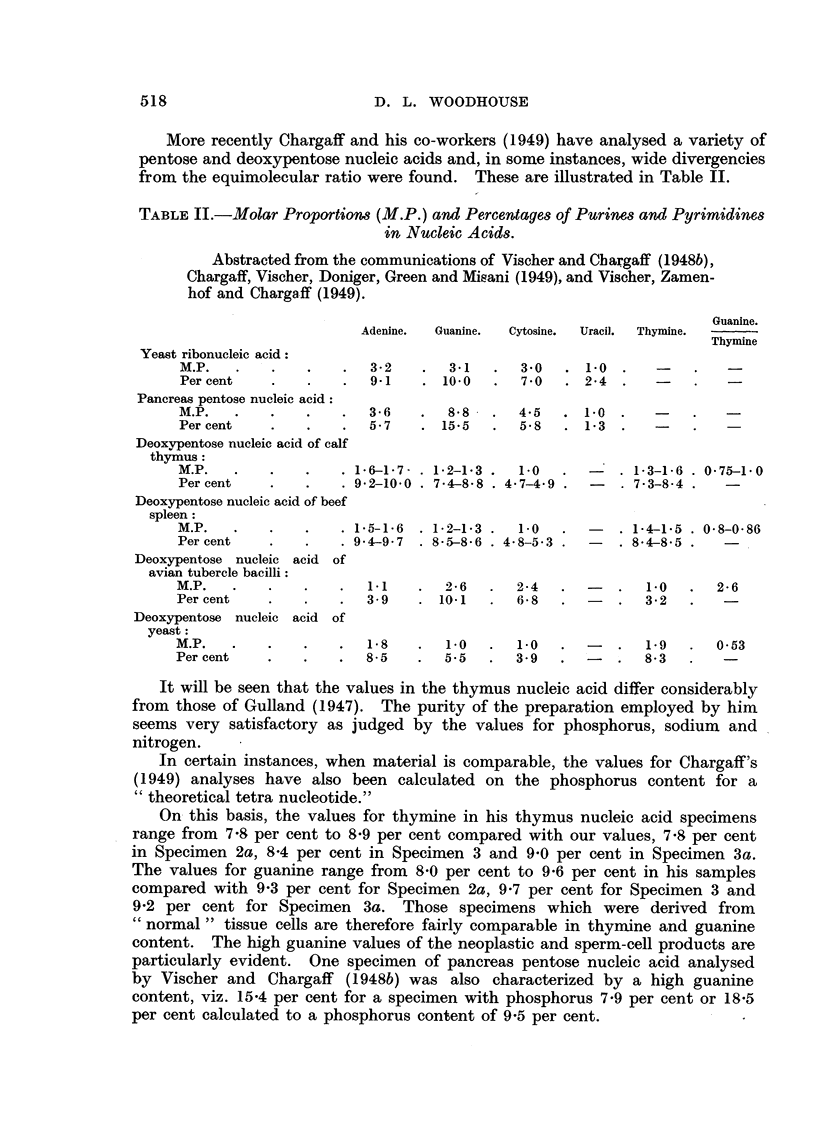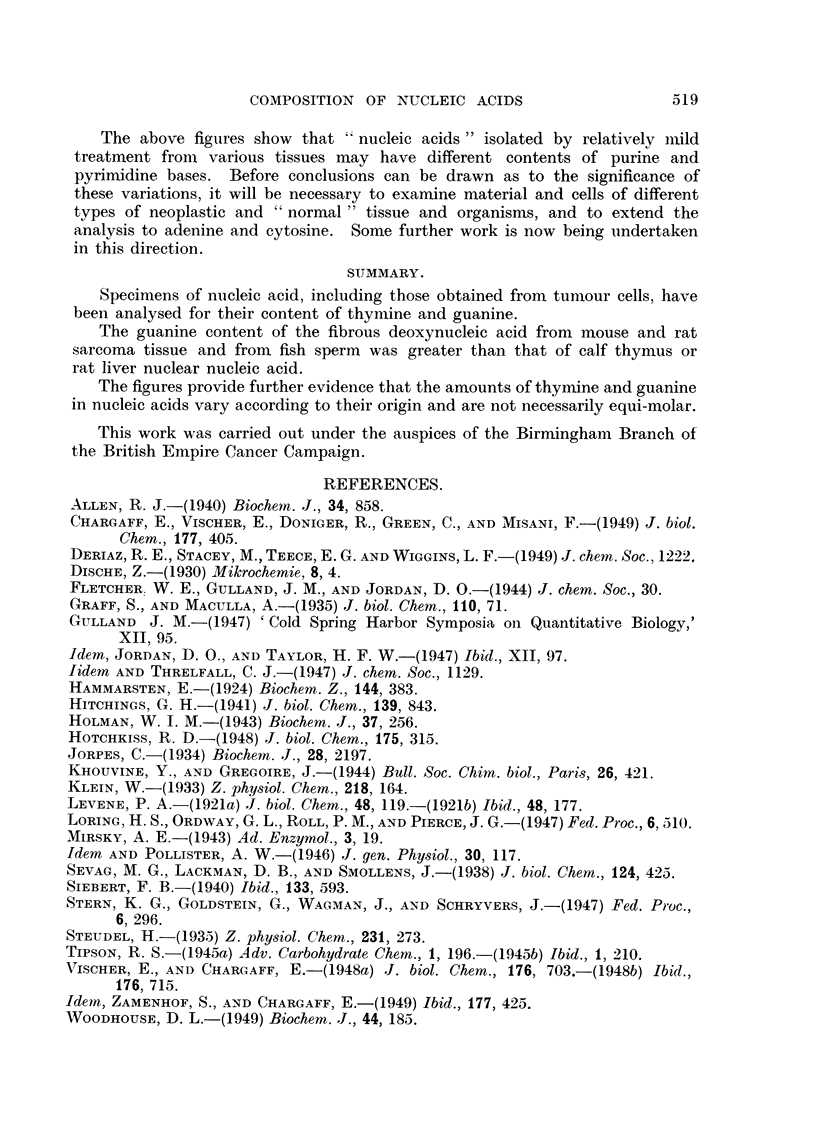# The Composition of Nucleic Acids: The Guanine and Thymine Content of Nucleic Acids Isolated from Normal Tissues and Animal Tumours

**DOI:** 10.1038/bjc.1949.54

**Published:** 1949-12

**Authors:** D. L. Woodhouse


### THE COMPOSITION OF NUCLEIC ACIDS: THE GUANINE AND THYMINE CONTENT OF NUCLEIC ACIDS ISOLATED FROM NORMAL TISSUES AND ANIMAL TUMOURS.

D. L. WOODHOUSE.

*From the Cancer Research Laboratory, Medical School, University of Birmingham, Edgbaston, Birmingham, 15.*

Received for publication October 12, 1949.

BECAUSE it is recognized that nucleic acids are macro-molecular in structure it is necessary to examine critically the analytical data upon which the idea of a tetra-nucleotide arrangement is based. Tipson (1945*a*) stated, " On hydrolysing ribosenucleic acid with very dilute ammonia at 115° for 1 hour, or with cold alkali, there is obtained an *equimolecular* mixture of four nucleotides." Also (Tipson, 1945*b*), " Since mild alkaline hydrolysisof ribose nucleic acid gives directly an *equimolecular* mixture of the four mononucleotides, formulas proposed for ribosenucleic acid . . . will be ignored unless they show the union of four mononucleotides."

Evidence has accumulated, however, which suggests that the simple tetra-nucleotide formula of Levene (1921*a*) for deoxy-ribonucleic acids, and similar formulae requiring simple repeating polynucleotide units, do not express accurately the composition of these substances derived from calf thymus or spleen (Chargaff, Vischer, Doniger, Green and Misani, 1949), or for the deoxy-pentose nucleic acid from avian tubercle bacilli or from yeast (Vischer, Zamenhof and Chargaff, 1949). It appears equally probable that the analogous structure applied to pentose nucleic acid may also need revision (Loring, Ordway, Roll and Pierce, 1947).

Gulland (1947) emphasized the fact that four basic factors determine the structure of the polynucleotides: the nature of the individual nucleotides, the nature of the linkages between the nucleotides, the relative positions of the nucleotides and the relative proportions of these nucleotides ; and that not one of these factors is known with complete certainty for any one polynucleotide.

In order to provide evidence regarding the composition of such compounds it is necessary to prepare the nucleic acid from various sources, under conditions which are least likely to cause decomposition or degradation, and to determine the units by accurate and specific methods of analysis. If analyses can be made for the constituent units by methods which do not necessitate their complete isolation, errors inherent in such separations would be avoided. The techniques of Hotchkiss (1948) for chromatographic isolation of the purines and pyrimidines from hydrolysed nucleic acids, and also those of Vischer and Chargaff (1948*a*), and Chargaff *et al.* (1949), are notable contributions in this field of work. The latter combined controlled hydrolysis and chromatographic separation of the bases, followed by quantitative estimation of the purines and pyrimidines by ultra-violet absorption spectrometry.

Work has been carried out in this laboratory to obtain data concerning the relative proportions of the purines and pyrimidines in nucleic acids, particularly with respect to the content of thymine and guanine, and results obtained with a series of nucleic acids, including specimens of deoxyribose nucleic acid from animal tumours, are described in this communication. Values for the nuclear nucleic acids of tumours have not previously been reported.

Absorption colorimetry has been employed for the estimations of these bases, and the methods, therefore, afford data complementary to those given by ultra-violet absorption.

### MATERIALS.

Fourteen specimens of nucleic acid were utilized in these studies.

*Specimens* 1 *and* 2 were commercial yeast ribose nucleic acid and commercial thymus nucleic acid respectively in the form of sodium salts. Samples 1*a* and 2*a* were derived from these by purification in a manner similar to that of Fletcher, Gulland and Jordan (1944) by precipitations from alcohol acidified with acetic acid.

*Specimen* 3 was prepared in the laboratory from fresh calf thymus according to the method of Hammarsten (1924). Specimen 3*a* was extracted from similar material by the method of Mirsky and Pollister (1946).

*Specimens* 4 *and* 4*a* were derived from transplanted mouse sarcoma tissue. The tumour was originally induced by intra-muscular injection of 1:2:5:6-dibenzanthracene, and has been transmitted during the course of 12 years through about 270 generations. It is a spindle-celled sarcoma with little stroma. Non-necrotic tumours weighing 4 to 6 g. are obtained after 10 to 14 days in about 80 per cent of the animals inoculated. To extract a specimen of nucleoprotein the tumours from 30 mice were minced in a Waring blendor with 200 ml. of cold physiological saline containing M/1000 sodium arsenate as enzyme inhibitor. The cytoplasmic nucleoprotein is extracted by this procedure and remains in the supernatant liquid when the extract is centrifuged at moderate speed. This was discarded and the operation repeated several times, thus effecting a considerable removal of cytoplasmic protein.

The residue was then extracted for several hours by stirring with M saline when a viscous solution resulted. This was centrifuged to remove insoluble debris, and the nucleoprotein in the supernatant fluid was further purified by repeated precipitations in diluted saline as described by Mirsky and Pollister (1946).

From this nucleoprotein the nucleic acid was separated by the chloroform-octyl alcohol method of Sevag, Lackman and Smollens (1938). The process was repeated four times for Specimen 4*a*.

Fibrous, white preparations were obtained and solutions containing 0·5 per cent were strongly birefringent. Further purification by the method of alcohol precipitation was effected for Specimen 4*a*.

*Specimen* 5.—Deoxypentose nucleic acid was prepared similarly from transplanted rat sarcomas.

*Specimens* 6 *and* 6*a* were obtained from livers of untreated rats and from those bearing transplanted sarcomas by extracting the " nuclear " nucleic acid using the above procedure.

*Specimen* 7 was deoxyribose nucleic acid obtained by a similar procedure from human spleen.

*Specimens* 8 *and* 9.—These were kindly provided by Dr. F. C. Li, of the Chemistry Department, University of Birmingham. They consisted of a purified specimen of fibrous fish-sperm nucleic acid, and a specimen derived from the original nucleic acid by procedures used in his studies on the structure of nucleic acid.

### METHODS.

Analyses for phosphorus content, deoxypentose, thymine and guanine were carried out on these samples, using the following methods of colorimetric determination in which a Spekker absorptiometer was employed.

*Phosphorus estimation.*

The method of Holman (1943) was very satisfactory for determining the phosphorus content in hydrolysates obtained by the method of Allen (1940). The blue colour was stable for many hours and replicate analysis gave values differing by not more than 2 per cent for solutions containing between 20 and 100 $\mu$g. phosphorus in 2 ml., the final volume for colorimetry being 25 ml. The colour intensity in this range followed Beer's Law very closely when either of the colour filters, Chance OR 2 or Ilford 608, was used.

*Nucleic acid value by deoxypentose test.*

Comparative values for the different samples were obtained by the diphenylamine test of Dische (1930). This does not yield absolute values for the pentose content, and it seems probable that under the conditions of the test the colours produced by the purine nucleotides are not the same as those obtained by equimolecular amounts of pyrimidine nucleotides. For analyses of nucleic acids a standard interaction time of 15 minutes in boiling water was used and the colours were compared using an Ilford green filter H604. A longer period of reaction with the diphenylamine solution at 100° C. somewhat increases the colour intensity. The colour also increases slowly on standing at room temperature so that accurate timing is necessary. It was found necessary to purify the diphenylamine by twice recrystallizing commercial specimens from petroleum ether. Acetic acid solutions of the reagent thus purified remained colourless over long periods, while " blank " tests gave only a very slight colour.

The chart obtained by plotting " colour produced " against the " quantity of deoxyribose nucleic acid present " in a final volume of 10 ml. of solution was slightly curved between the limits 0·50 and 2·0 mg., but this was reproducible under the standard conditions. The curve obtained with pure deoxyribose under similar conditions (0·10 to 0·50 mg. in final volume of 10 ml.) is similar in type, but the intensity of colour obtained from a known weight of pentose is greater than that produced from an equivalent amount of deoxyribose nucleic acid if this is calculated assuming the conventional tetra-nucleotide structure. This may be because some portion of the deoxyribose in the nucleic acid cannot readily take part in the reaction.

The Dische reaction has been studied by Deriaz, Stacey, Teece and Wiggins (1949). They compared the colour produced by deoxy derivatives and related compounds which give the characteristic blue colour, substances which give no interfering colour including glucose, ascorbic acid and certain aldehydes, those which give green colours—generally not of great intensity, e.g. aldol, glyceraldehyde, and hexals, which give pink violet tints. They concluded that for comparative results the presence of such substances as decomposition product during the interaction should not interfere with the test if carried out rigidly. In their experiments they heated for 3·25 minutes in the boiling water bath, in which time the colour development for nucleic acid is incomplete. As shown by experiments described later, for quantitative work it is necessary to hydrolyse for 15 minutes.

*Thymine estimation.*

The technique described by Woodhouse (1949) was followed in this investigation. Vischer and Chargaff (1948*b*) described a useful micro-scale apparatus for the first stage in the separation of the purines in which the nucleic acid is decomposed with a rapid stream of dry HCl while suspended in methanol. These workers carried out the subsequent step of decomposing the pyrimidine nucleotides in sealed tubes, using concentrated formic acid. In this work the hydrolyses were effected with 30 per cent wt./vol. sulphuric acid. Formic acid has some advantage because it does not decompose cytosine. This, however, was not under examination in the present investigation, while sulphuric acid was known not to interfere with subsequent colorimetry. Sulphuric acid at the temperature (175° C.) employed does not decompose thymine. It can be recovered by the colour reaction, after two hours' treatment with this acid under the conditions employed, without loss.

*Guanine estimation.*

The method used for estimating this compound colorimetrically was that described by Hitchings (1941). The reaction, which utilizes the Folin-Ciocalteau reagent, although not specific, is very sensitive for guanine. Adenine, hypoxanthine, thymine and cytosine give no colour. Xanthine yields an identical colour value per mol., while 2-oxy-6-amino-purine also gives a similar colour. However, these latter purine compounds were not encountered in the hydrolysates of the samples of nucleic acid which were examined by Vischer and Chargaff (1948 ) by chromatographic technique and do not appear to be present in nucleic acid.

The method of developing colour with this reagent is simple and speedy, and it can be used accurately over a range of 20 to 150 μg. guanine in a final volume of 10 ml. The colour value follows Beer's Law very closely. The blue colour is very stable, and can be determined in the Spekker Absorptiometer without a filter.

It was necessary to ascertain whether any substances present in the hydrolysis products of nucleic acid would interfere with the accurate analysis of the guanine. The following tests were made during the course of various experiments undertaken to investigate the conditions necessary for the determination of the guanine content of nucleic acid and nucleotides.

The figures given in these and subsequent analyses are means of at least two duplicate experiments made on two initial samples.

A. *Recovery from mixed purines.*—From a solution containing 0·100 mg. thymine, 0·100 mg. guanine and 0·200 mg. adenine, the purines could be precipitated with silver acetate and the guanine determined quantitatively by the Hitchings' colorimetric procedure. From the remaining fluid the pyrimidine-

34

silver salt could be precipitated by making alkaline with barium hydroxide, and by proceeding with the method for thymine (Woodhouse, 1949) a quantitative recovery as measured by the colour value for this component obtained.

B. *Recovery from guanylic acid.*—The guanine could be determined quantitatively in a solution of a pure sample of guanylic acid after hydrolysing with 5N sulphuric acid.

10 mg. guanylic acid were dissolved in 25 ml. distilled water.

(1) 5 ml. were refluxed with 5 ml. 10N sulphuric acid for 2 hours and then made up to 10 ml.; 1·0 ml. of this solution taken for colorimetric determination.

*Found* (in original 10 mg. guanylic acid) = 4·05 mg.

$$\text{Theoretical}\left(\frac{151}{382}\times 10\text{ mg.}\right) = 4{\cdot}0\text{ mg.}$$

(2) 5 ml. of the hydrolysed solution were nearly neutralized with sodium carbonate, silver acetate was added and the silver purine centrifuged, decomposed with 2 ml. N hydrochloric acid and made up to 10 ml. 2·0 ml. of this solution were taken for the estimation and made up to 10 ml.

*Found* (for original 10 mg. guanylic acid) = 4·04 mg.

C. *Control tests.*

(1) 10 mg. adenylic acid after hydrolysis gave by direct application of the F.C. reagent a colour equal to only 1·4 $\mu$g. of guanine. Thus the hydrolysis products, e.g. adenine or products from ribose, gave a negligible colour value.

(2) 2 ml. pure deoxyribose (provided by Professor Stacey, Chemistry Department, University of Birmingham) containing 1·6 mg. pentose gave a negligible colour with the F.C. reagent, the colour value being equal to 11 $\mu$g.

(3) 2·0 mg. guanylic acid contained in 5 ml. dilute HCl were hydrolysed with the Formic acid-HCl mixture (N HCl and 10 N HCOOH) of Graff and Maculla (1935) for 2 hours under reflux. 5 ml. of the mixture were made up to 25 ml., of which 2 ml. were taken for test.

Guanine found . . . . = 61·0 $\mu$g.

Theoretical value of guanine in this = 63·6 $\mu$g.

(4) No colour was produced with the F.C. reagent by 10 times the amount of formic acid employed in the above test.

(5) A solution containing 1·0 mg. guanine in 20 ml. was refluxed for 2 hours with the concentration of formic HCl acid used in these tests. When aliquots of the diluted solution were tested they yielded theoretical colour, proving that no destruction of guanine took place.

### RESULTS.

The values for the phosphorus, thymine and guanine content of the specimens and their colour production by the diphenylamine test compared with commercial thymus nucleic acid are given in Table I. The spleen nucleic acid gave a low value for phosphorus and guanine. This may be due possibly to autolytic changes in the spleen which was obtained post mortem. The nucleic acid was

TABLE I.—*Results.*

| | Phosphorus content (%). | Dische value. | Thymine content (%). | Guanine content (%). | Adjusted thymine (%). | Adjusted guanine (%). | Guanine thymine molar ratio. |
|---|---|---|---|---|---|---|---|
| 1. Commercial yeast nucleic acid sodium salt | 7·4 | — | — | 10·4 | — | — | — |
| 1*a*. Ditto purified | 7·8 | — | — | 10·0 | — | — | — |
| 2. Commercial calf thymus | 7·3 | 100 | 6·4 | 8·1 | 8·0 | 10·2 | 0·96 |
| 2*a*. Ditto purified (free acid) | 8·3 | 103 | 7·0 | 8·4 | 8·4 | 10·7 | 0·95 |
| 3. Calf thymus nucleic acid sodium salt (Hammarsten) | 7·6 | 115 | 7·0 | 8·0 | 8·5 | 9·8 | 0·875 |
| 3*a*. Ditto (Mirsky) | 8·4 | 125 | 9·0 | 8·4 | 9·9 | 9·2 | 0·80 |
| 4. Mouse sarcoma nucleic acid sodium salt | 7·8 | 109 | 9·0 | 13·0 | 10·7 | 15·4 | 1·08 |
| 4*a*. Ditto | 8·3 | 114 | 9·0 | 13·8 | 10·0 | 15·2 | 1·14 |
| 5. Rat sarcoma nucleic acid sodium salt | 8·4 | 127 | 8·9 | 14·7 | 9·8 | 16·1 | 1·23 |
| 6. Nucleic acid from livers of tumour-bearing rats | 7·5 | 81 | 7·8 | 10·1 | 9·7 | 12·6 | 0·98 |
| 6*a*. Nucleic acid from normal rat liver | 7·4 | 77 | 7·4 | 9·6 | 9·2 | 12·0 | 0·98 |
| 7. Nucleic acid from human spleen | 6·5 | 102 | 5·3 | 9·1 | 7·5 | 12·9 | 1·30 |
| 8. Nucleic acid from fish sperm | 8·75 | 121 | 7·75 | 14·4 | 7·8 | 14·6 | 1·46 |
| 9. Nucleic acid A | 9·8 | 138 | 7·8 | 14·8 | 7·9 | 15·0 | 1·43 |
| "Theoretical" ribose nucleic acid | 9·5 | — | — | 11·5 | — | — | — |
| "Theoretical" ribose nucleic acid tetra sodium salt | 8·9 | — | — | 10·8 | — | — | — |
| "Theoretical" deoxyribose nucleic acid | 9·9 | — | 9·1 | 12·0 | — | — | 1·0 |
| "Theoretical" deoxyribose nucleic acid tetra sodium salt | 9·25 | — | 8·5 | 11·3 | — | — | 1·0 |

found to contain a small amount of protein impurity, about 3 per cent, but this could not account entirely for the low phosphorus value. The phosphorus content of the other tissue nucleic acid specimens varied from 7·3 to 8·4 per cent, while the sample of sperm nucleic acid had 8·75 per cent phosphorus and the nucleic acid A practically the theoretical value of 9·8 per cent.

The thymine content was low in the commercial specimens and highest in the sarcoma nucleic acids, while the guanine values were particularly high in the tumour-derived specimens. The values for these units have also been recalculated to ascertain the percentage which would be present if the phosphorus content of the various specimens had been that of the conventional tetranucleotide, i.e. 9·5 per cent for ribose nucleic acid, 9·8 per cent for the deoxyribose nucleic acid and 9·25 per cent for the tetra sodium salt. These values are given in columns 5 and 6 in Table I. The theoretical content of thymine and guanine for a "pure" tetranucleotide nucleic acid are also given. Whereas the thymine values found in our specimens are fairly similar to the "theoretical" figure, the guanine contents of the tumour nucleic acid and the sperm nucleic acid are 25 per cent to 50 per cent above the "theoretical" amount.

It will be observed that on this basis the thymine content varies from 7·8 per cent to 12·0 per cent and the guanine from 9·2 per cent to 16·1 per cent. The high values for guanine in the sarcoma-derived specimens may be of special note.

DISCUSSION.

In appraising the significance of the analytical results the " purity " of the preparations is a major consideration. It is very difficult to assess the quality of a macro-molecular substance or fraction whose structure and composition are under review, since none of the contained elements or units can be accepted as a suitable criterion.

Commercial preparations, and those isolated by methods involving treatment with reactive chemicals, may contain degraded types and decomposition products. Many of the samples prepared for the present investigation were isolated by techniques which are believed to minimize such decomposition ; in some instances an enzyme inhibitor in the form of M/1000 sodium arsenate was added to the initial stages of the isolation of the nucleoprotein with the object of preventing cleavage by nucleo-phosphatases (Klein, 1933) or by depolymerases (Stern, Goldstein, Wagman and Schryvers, 1947).

The complete removal of the protein from the nucleic acid requires special attention, particularly in preparations made from liver and from spleen, and experience showed that the process of dissociation by the chloroform-octyl alcohol method of Sevag, Lackman and Smollens (1938) must be repeated many times to remove the protein completely. Khouvine and Gregoire (1944) recommended as many as 30 treatments.

*Phosphorus content.*

Even with these precautions the phosphorus content appears to fall short of that required by the conventional formulation of nucleic acid ; the values range from 7·6 per cent. to 8·4 per cent. (the value for nucleic acid A was exceptional, and may be due to the method of preparation), whereas the required figure is 8·9 per cent for the ribose tetra sodium salt and 9·2 per cent for the tetra sodium salt of deoxyribose nucleic acid.

This " low " phosphorus content has also been characteristic of the preparations described by many other workers, e.g. Hammarsten (1924) 7·09 per cent., Jorpes (1934) 5·7 per cent for the barium salt (theoretical 7 per cent), Loring *et al.* (1947) 8·27 per cent to 8·77 per cent for commercial yeast nucleic acids, 8·2 per cent for tobacco virus nucleic acid and 7·78 per cent to 8·3 per cent for certain fractions of yeast nucleic acid. Also the yeast ribose nucleic acid analysed by Vischer and Chargaff (1948*b*), which was purified from commercial material in a similar manner to that used for Specimens 1 and 2 in this paper, had 8·0 per cent phosphorus, while the material they obtained from pig pancreas had 7·9 per cent. The deoxyribose nucleic acid from calf thymus obtained by Chargaff *et al.* (1949) using the Hamarsten (1924) method contained 8·0 per cent, while a similar content was present in a specimen purified via the lanthanum salt and converted to the potassium salt which was used as a standard for the determinations of the deoxypentose content of other specimens. Specimens isolated by these workers by the method of Gulland, Jordan and Threlfall (1947) contained only 7·6 per cent. Beef spleen deoxypentose nucleic acids isolated by the Mirsky and Pollister (1946) process, in which the protein was removed by 9 treatments of a solution of nucleoprotein in physiological saline with chloroform-octyl alcohol, appeared to be of high purity, as measured by the phosphorus content, which was 8·6 to 8·8 per cent. On the other hand, from avian tubercle bacilli a nucleic acid was isolated, which, after 5 treatments with chloroform-octyl alcohol mixture each of 16 hours' duration, contained only 4·8 per cent phosphorus. By the action of deoxycholate on the nucleo-protein, and subsequent chloroform-octyl alcohol treatment and other purification steps, a specimen containing 6·3 per cent phosphorus was obtained. By electrophoresis a component with 7·6 per cent phosphorus was produced from a crude nucleic acid extracted from ether-washed bacilli with deoxycholic acid.

The above figures indicate the great difficulty in removing contaminating substances from undegraded nucleic acids, but it is possible that some of the variation may be actual and not due to " impurity."

*The content of purines and pyrimidines.*

Some early reports suggested that the amounts of those compounds obtained by hydrolysis of nucleic acid were not consistent with the tetra-nucleotide theory (Levene, 1921*b*; Steudel, 1935).

Mirsky (1943), however, described the ratio of total purines to pyrimidine in deoxy-pentose nucleic acid as " equimolecular " in specimens obtained from animal tissues and from wheat germ. This conclusion was based partly on experimental evidence, which suggested that the ratio of purines to pyrimidines in nucleic acids could be calculated by comparing the intensity of colour obtained by the Dische diphenylamine reaction (Dische, 1930) with the total nucleic acid as indicated by the phosphorus content. It was believed that the blue diphenylamine colour was produced almost entirely by the purine pentose, and that the contribution of the pyrimidine nucleotide was negligible.

Experiments have been carried out in this laboratory comparing the colours produced by deoxyribose and deoxyribose nucleic acid with the Dische reagent when the standard reaction time of 15 minutes (Siebert, 1940) was used. Also curves were drawn comparing the colour produced after various times of hydrolysis from 5 to 40 minutes, by the isolated sugar and by nucleic acid. The colour development was rapid during the first 15 minutes, and continued at a slower rate for at least 40 minutes. The form of the curves obtained by plotting time against colour for the deoxypentose was the same as that for the nucleic acid. However, on calculating the total amount of pentose which would be contained in a given weight of nucleic acid (assuming a tetranucleotide of conventional structure), the amount of colour obtained in these tests was less than that produced by the equivalent amount of free deoxyribose, but greater than would be produced if the colour were contributed by the purine-combined portion only. It was concluded that the proportions of purine and pyrimidine nucleotides could not be accurately calculated by means of this reaction.

Gulland, Jordan and Taylor (1947) obtained values by electrometric titrations of a specimen of calf thymus deoxypentose nucleic acid sodium salt of high grade (phosphorus, 9·33 per cent ; nitrogen, 15·3 per cent ; sodium, 6·9 per cent), which indicated that the proportions were 1 radical of guanine, 1 of thymine, 1·2 cytosine and 0·8 adenine. Also, when analyses were conducted on the purine and pyridmidine fractions obtained from the hydrolysate of a sample of yeast nucleic acid by precipitations with silver acetate, results were obtained suggesting that the ratio of purine to pyrimidine nitrogen was 1·86 instead of the theoretical 2·0 for a statistical tetra nucleotide.

More recently Chargaff and his co-workers (1949) have analysed a variety of pentose and deoxypentose nucleic acids and, in some instances, wide divergencies from the equimolecular ratio were found. These are illustrated in Table II.

TABLE II.—*Molar Proportions (M.P.) and Percentages of Purines and Pyrimidines in Nucleic Acids.*

Abstracted from the communications of Vischer and Chargaff (1948*b*), Chargaff, Vischer, Doniger, Green and Misani (1949), and Vischer, Zamenhof and Chargaff (1949).

| | Adenine. | Guanine. | Cytosine. | Uracil. | Thymine. | Guanine. / Thymine |
|---|---|---|---|---|---|---|
| Yeast ribonucleic acid : | | | | | | |
| M.P. | 3·2 | 3·1 | 3·0 | 1·0 | — | — |
| Per cent | 9·1 | 10·0 | 7·0 | 2·4 | — | — |
| Pancreas pentose nucleic acid : | | | | | | |
| M.P. | 3·6 | 8·8 | 4·5 | 1·0 | — | — |
| Per cent | 5·7 | 15·5 | 5·8 | 1·3 | — | — |
| Deoxypentose nucleic acid of calf thymus : | | | | | | |
| M.P. | 1·6–1·7 | 1·2–1·3 | 1·0 | — | 1·3–1·6 | 0·75–1·0 |
| Per cent | 9·2–10·0 | 7·4–8·8 | 4·7–4·9 | — | 7·3–8·4 | — |
| Deoxypentose nucleic acid of beef spleen : | | | | | | |
| M.P. | 1·5–1·6 | 1·2–1·3 | 1·0 | — | 1·4–1·5 | 0·8–0·86 |
| Per cent | 9·4–9·7 | 8·5–8·6 | 4·8–5·3 | — | 8·4–8·5 | — |
| Deoxypentose nucleic acid of avian tubercle bacilli : | | | | | | |
| M.P. | 1·1 | 2·6 | 2·4 | — | 1·0 | 2·6 |
| Per cent | 3·9 | 10·1 | 6·8 | — | 3·2 | — |
| Deoxypentose nucleic acid of yeast : | | | | | | |
| M.P. | 1·8 | 1·0 | 1·0 | — | 1·9 | 0·53 |
| Per cent | 8·5 | 5·5 | 3·9 | — | 8·3 | — |

It will be seen that the values in the thymus nucleic acid differ considerably from those of Gulland (1947). The purity of the preparation employed by him seems very satisfactory as judged by the values for phosphorus, sodium and nitrogen.

In certain instances, when material is comparable, the values for Chargaff's (1949) analyses have also been calculated on the phosphorus content for a "theoretical tetra nucleotide."

On this basis, the values for thymine in his thymus nucleic acid specimens range from 7·8 per cent to 8·9 per cent compared with our values, 7·8 per cent in Specimen 2*a*, 8·4 per cent in Specimen 3 and 9·0 per cent in Specimen 3*a*. The values for guanine range from 8·0 per cent to 9·6 per cent in his samples compared with 9·3 per cent for Specimen 2*a*, 9·7 per cent for Specimen 3 and 9·2 per cent for Specimen 3*a*. Those specimens which were derived from "normal" tissue cells are therefore fairly comparable in thymine and guanine content. The high guanine values of the neoplastic and sperm-cell products are particularly evident. One specimen of pancreas pentose nucleic acid analysed by Vischer and Chargaff (1948*b*) was also characterized by a high guanine content, viz. 15·4 per cent for a specimen with phosphorus 7·9 per cent or 18·5 per cent calculated to a phosphorus content of 9·5 per cent.

The above figures show that "nucleic acids" isolated by relatively mild treatment from various tissues may have different contents of purine and pyrimidine bases. Before conclusions can be drawn as to the significance of these variations, it will be necessary to examine material and cells of different types of neoplastic and "normal" tissue and organisms, and to extend the analysis to adenine and cytosine. Some further work is now being undertaken in this direction.

### SUMMARY.

Specimens of nucleic acid, including those obtained from tumour cells, have been analysed for their content of thymine and guanine.

The guanine content of the fibrous deoxynucleic acid from mouse and rat sarcoma tissue and from fish sperm was greater than that of calf thymus or rat liver nuclear nucleic acid.

The figures provide further evidence that the amounts of thymine and guanine in nucleic acids vary according to their origin and are not necessarily equi-molar.

This work was carried out under the auspices of the Birmingham Branch of the British Empire Cancer Campaign.

### REFERENCES.

ALLEN, R. J.—(1940) *Biochem. J.*, **34**, 858.

CHARGAFF, E., VISCHER, E., DONIGER, R., GREEN, C., AND MISANI, F.—(1949) *J. biol. Chem.*, **177**, 405.

DERIAZ, R. E., STACEY, M., TEECE, E. G. AND WIGGINS, L. F.—(1949) *J. chem. Soc.*, 1222.

DISCHE, Z.—(1930) *Mikrochemie*, **8**, 4.

FLETCHER, W. E., GULLAND, J. M., AND JORDAN, D. O.—(1944) *J. chem. Soc.*, 30.

GRAFF, S., AND MACULLA, A.—(1935) *J. biol. Chem.*, **110**, 71.

GULLAND J. M.—(1947) 'Cold Spring Harbor Symposia on Quantitative Biology,' XII, 95.

*Idem*, JORDAN, D. O., AND TAYLOR, H. F. W.—(1947) *Ibid.*, XII, 97.

*Iidem* AND THRELFALL, C. J.—(1947) *J. chem. Soc.*, 1129.

HAMMARSTEN, E.—(1924) *Biochem. Z.*, **144**, 383.

HITCHINGS, G. H.—(1941) *J. biol. Chem.*, **139**, 843.

HOLMAN, W. I. M.—(1943) *Biochem. J.*, **37**, 256.

HOTCHKISS, R. D.—(1948) *J. biol. Chem.*, **175**, 315.

JORPES, C.—(1934) *Biochem. J.*, **28**, 2197.

KHOUVINE, Y., AND GREGOIRE, J.—(1944) *Bull. Soc. Chim. biol., Paris*, **26**, 421.

KLEIN, W.—(1933) *Z. physiol. Chem.*, **218**, 164.

LEVENE, P. A.—(1921*a*) *J. biol. Chem.*, **48**, 119.—(1921*b*) *Ibid.*, **48**, 177.

LORING, H. S., ORDWAY, G. L., ROLL, P. M., AND PIERCE, J. G.—(1947) *Fed. Proc.*, **6**, 510.

MIRSKY, A. E.—(1943) *Ad. Enzymol.*, **3**, 19.

*Idem* AND POLLISTER, A. W.—(1946) *J. gen. Physiol.*, **30**, 117.

SEVAG, M. G., LACKMAN, D. B., AND SMOLLENS, J.—(1938) *J. biol. Chem.*, **124**, 425.

SIEBERT, F. B.—(1940) *Ibid.*, **133**, 593.

STERN, K. G., GOLDSTEIN, G., WAGMAN, J., AND SCHRYVERS, J.—(1947) *Fed. Proc.*, **6**, 296.

STEUDEL, H.—(1935) *Z. physiol. Chem.*, **231**, 273.

TIPSON, R. S.—(1945*a*) *Adv. Carbohydrate Chem.*, **1**, 196.—(1945*b*) *Ibid.*, **1**, 210.

VISCHER, E., AND CHARGAFF, E.—(1948*a*) *J. biol. Chem.*, **176**, 703.—(1948*b*) *Ibid.*, **176**, 715.

*Idem*, ZAMENHOF, S., AND CHARGAFF, E.—(1949) *Ibid.*, **177**, 425.

WOODHOUSE, D. L.—(1949) *Biochem. J.*, **44**, 185.